# Leptin and Metabolic Programming

**DOI:** 10.3390/nu14010114

**Published:** 2021-12-28

**Authors:** Catalina Picó, Mariona Palou

**Affiliations:** 1 Laboratory of Molecular Biology, Nutrition and Biotechnology (Nutrigenomics, Biomarkers and Risk Evaluation), University of the Balearic Islands, 07122 Palma, Spain; mariona.palou@uib.cat; 2 Health Research Institute of the Balearic Islands (IdISBa), 07122 Palma, Spain; 3 CIBER de Fisiopatología de la Obesidad y Nutrición (CIBEROBN), 07122 Palma, Spain

This Special Issue of *Nutrients* “Leptin and Metabolic Programming” includes one review article regarding the function of leptin throughout the entire life on cardiometabolic fates and four original articles related to the new function of leptin present in milk and liquid amniotic, its possible relation with other components of breast milk, and how environmental conditions may impact on leptin action and metabolic programming.

Leptin was originally described as an adipocyte-derived hormone, produced in proportion to the size of fat stores, and was regarded as a unique regulator of food intake and energy homeostasis [1]. Now, we know that leptin is also involved in many other functions, including glucose homeostasis, haematopoiesis, immune function, and reproduction and development [2]. Moreover, although the white adipose tissue is the primary source of circulating leptin, this hormone is also produced by other tissues, such as the stomach, playing a potential role in the short-term control of food intake as a satiety signal [3], but also the placenta and the mammary gland, and is present in breast milk [4]. This has further spread the interest in this hormone and has opened an interesting field of research regarding its long-lasting effects in metabolic programming and development.

Prenatal and early postnatal periods are sensitive life stages of development, and nutritional and environmental factors during such a window of plasticity may have a marked influence on later health [5]. Regarding lactation, there is clear evidence of the importance of breastfeeding, compared to formula feeding, in the protection against chronic diseases, particularly obesity and diabetes, and, although more modestly, on cardiovascular risk factors [6]. Interestingly, leptin is present in breast milk, but not in infant formula, and direct evidence from animal studies and indirect evidence from epidemiological studies point to the beneficial effects of the intake of leptin during this period in the programming of later metabolic health [4].

What is the function of leptin during lactation? Animal studies have revealed that leptin exerts key biological actions during lactation. It has an essential neurotrophic role and is important for the development of hypothalamic circuits involved in the control of energy homeostasis [7], therefore affecting the susceptibility to later overweight/obesity [4]. In rodents, this activity is restricted to a precise period of lactation, during the second postnatal week, in which there is a transient increase in plasma leptin levels, the so-called leptin surge [8]. The absence of such a transient increase in plasma leptin has been associated with an impairment in the neuronal organization of the centers engaged in the regulation of energy metabolism and food intake, therefore affecting the ability to adequately control energy balance and body weight in adulthood [9].

Supporting the key biological role of leptin during lactation, several interventional studies in rodents have concluded that leptin intake at physiological doses during the suckling period protects against excess fat accumulation and the development of metabolic disturbances in adulthood [4]. Concretely, neonate rats that were supplemented with leptin during the suckling period showed increased central and peripheral leptin sensitivity [10,11], and were more protected against the development of insulin resistance [12] and hepatic lipid accumulation when exposed to an obesogenic diet [11]. Notably, leptin supplementation has also been shown to ameliorate the dysmetabolic phenotype in the offspring associated with a mild/moderate gestational calorie restriction and exacerbated by exposure to a high-fat, high-sucrose diet (the so-called Western diet) in adulthood, which includes excess fat accumulation, adipose tissue inflammation, hypertriglyceridemia, hepatic steatosis, and insulin resistance [13]. These effects of leptin may also be attributed, at least in part, to its neurotrophic action during the suckling period, which is relevant since it conditions an adequate response to this hormone in later stages and allows a better homeostatic adaptation to environmental conditions.

Interestingly, one of the articles of this Special Issue shows that some of the neurotrophic effects observed with leptin supplementation during the suckling period in the offspring of dams exposed to a mild/moderate calorie restriction during gestation are mimicked, to some extent, by myo-inositol supplementation [14]. Myo-inositol is a component of breast milk with a potential interest during development. Myo-inositol supplementation at physiological doses during the suckling period in neonate rats had previously been shown to ameliorate insulin resistance and hypertriglyceridemia programmed by 25% gestational calorie restriction, comparable to leptin action [15]. In the present study, by analyzing the effects in young rats of the supplementation with physiological doses of both myo-inositol and leptin, separately and together, during the suckling period, it was shown that myoinositol can also reverse some of the alterations in hypothalamic structure and function programmed by maternal exposure to mild/moderate caloric restriction during gestation, in a sex-dependent manner. Such effects were comparable, to a certain extent, to those of leptin, and may account for the beneficial effects on the adult phenotype [15]. How myo-inositol exerts these effects is not yet established but considering that myoinositol supplemented animals displayed higher expression levels of the leptin receptor (*Lepr*) in the hypothalamus, it was hypothesized that myo-inositol may increase the sensitivity to leptin naturally ingested with milk [14]. It would be of interest to further explore this possibility as well as if other compounds may also empower leptin action during this critical period of development.

Being aware of the important function of leptin as a neurotrophic factor that contributes to the development of the feeding circuits in the arcuate nucleus of the hypothalamus (ARC), in another article of this monographic [16], the authors have evaluated whether postnatal overnutrition, which is known to affect leptin secretion and sensitivity, produces changes in the development of the synaptic transmission to the ARC in female mice. Postnatal overnutrition was achieved by raising mice in small litters and compared to animals raised in normal size litters. At the prepubertal stage, these animals showed higher body weight and subcutaneous adiposity and increased serum leptin levels than their controls. The authors show that the transition from prepubertal to the pubertal stage was characterized by a rise in both excitatory and inhibitory transmission to LepR-expressing neurons located in the ARC in control mice. Notably, postnatal overnutrition influenced the neuroplasticity of the ARC, since it induced a further increase in the excitatory synaptic transmission to LepR-expressing neurons in pubertal and adult animals, whereas the amplitude of inhibitory currents was reduced. Postnatal overnutrition also affected the expression levels of genes coding for ion channel receptors that play a role in neuronal plasticity and dendritic development in the ARC. Therefore, increased adiposity in early postnatal stages due to overfeeding during the suckling period has permanent effects on cellular excitability in the ARC, also affecting age-dependent changes in synaptic transmission, which corroborates the importance of this period of plasticity in the establishment of adequate neuronal connections.

In addition to leptin, breast milk contains other bioactive compounds with immunological properties that are transferred to the infant and may also be relevant in early life development. The profile of some of these compounds, particularly adipokines, including leptin, but also adiponectin and fibroblast growth factor 21 (FGF21), growth factors, such as epidermal growth factor (EGF) and β2 and β3 isoforms of transforming growth factor (TGF), and the immunoglobulin isotypes IgG, IgA, and IgM, in milk and plasma of rat dams and their relationships during the whole lactation period have been established in another article included in this monographic [17]. The authors show that the pattern of these bioactive compounds varies during the lactation period, and some of them (IgM, IgG, and the three adipokines) showed positive correlations between their levels in milk and plasma. Moreover, positive correlations were also found between themselves in each compartment (e.g., leptin and adiponectin in milk in the first period of lactation, and leptin and IgM in milk in the second period of lactation). It will also be interesting to further explore the function of these compounds as breast milk components.

Besides the role of milk leptin, prenatal leptin also seems to be key in the regulation of many processes during pregnancy, including fetal growth and the development of several tissues [18]. However, leptin is present in different embryonic and extraembryonic compartments, and its regulation and effects on fetal development are not well established. In this regard, in another article of this monographic [19], the authors show that leptin appears suddenly in the amniotic fluid at gestational day 20 in rats, which seems to be related to changes in the localization of placental leptin, and then undergoes a marked increase on day 21. Interestingly, leptin present in the amniotic fluid seems to be internalized by the immature stomach of the fetus, when the amniotic fluid is swallowed, playing a potential physiological role in near-term fetuses regarding short-term feeding control and metabolic programming.

Finally, in the review article of this monographic [20], the authors update the knowledge on the temporal effects of leptin throughout the entire life (gestational, neonatal to early life, and adulthood periods) on cardiometabolic physiology. In addition to the effects of leptin in adulthood and the consequences of a disruption in central leptin signaling in the development of chronic metabolic diseases, the authors describe the effects of maternal leptin on the development and growth of the infant during gestation, the metabolic adaptations that occur in the mother leading to leptin induction during this period, and the potential effects of leptin on the cardiometabolic state at later stages. The benefits of the early intake of leptin at adequate amounts as a component of breast milk and the adverse effects of alterations in serum leptin levels in infants, e.g., associated with excess adiposity/body weight, as possible drivers of cardiometabolic alterations, are also discussed.

To summarize, leptin exerts a broad spectrum of short, medium, and long-term regulatory actions on different target tissues to control whole-body physiological homeostasis. Maternal leptin also plays a central role in the appropriate programming and development of the infant during gestation and lactation. It could be hypothesized that leptin ingested during the early stages of development, either swallowed with the amniotic fluid or ingested as a component of breast milk, may act on different targets and perform some different functions from those described for plasma leptin. Therefore, the effects of ingesting higher physiological amounts of leptin during early postnatal life might be expected to be different from those observed with the presence of greater plasma leptin levels due to increased adiposity and/or postnatal overnutrition. However, these aspects and the overall function of leptin during the early stages of development merit further exploration.
